# Insects as Diet and Therapy: Perspectives on Their Use for Combating Diabetes Mellitus in Tanzania

**DOI:** 10.3390/ph14121273

**Published:** 2021-12-06

**Authors:** Geert René Verheyen, Luc Pieters, Sheila Maregesi, Sabine Van Miert

**Affiliations:** 1 RADIUS, Thomas More University of Applied Sciences, Kleinhoefstraat 4, 2440 Geel, Belgium; sabine.vanmiert@thomasmore.be; 2 NatuRA, Department of Pharmaceutical Sciences, University of Antwerp, Universiteitsplein 1, 2610 Wilrijk, Belgium; luc.pieters@uantwerpen.be; 3 Pharmacognosy Department, School of Pharmacy, Muhimbili University of Health and Allied Sciences, Dar Es Salaam 65013, Tanzania; smaregesi@hotmail.com

**Keywords:** traditional medicine, entomotherapy, entomophagy, diabetes, insects

## Abstract

More than 450 million people worldwide are suffering from diabetes and this number is expected to increase. In developing countries, such as Tanzania, the number of patients suffering from diabetes and associated diseases is increasing as well. Up to 80% of the Tanzanian people rely on traditional medicines for their health care services. The nature of Tanzanian is very rich in different plant and insect species, and this could be exploited through their implementation in preventive and/or curative approaches in the battle against diabetes. The implementation of healthy insects in the diets of people may help in the prevention of obesity, which is a risk factor in the etiology of diabetes, while the identification of small molecules in insects may help in the discovery of potential new drugs that can be used in the treatment of diabetes. In this paper, an overview on the potential implementation of insects against diabetes is presented.

## 1. Introduction

Diabetes mellitus is a chronic disease that is characterized by raised blood glucose levels. This is caused by an insufficient production or inefficient use of the hormone insulin, which is produced by the pancreatic beta-cells and helps glucose from the blood enter the cells where it is converted into energy. If left untreated, diabetes can cause other problems, including cardiovascular and eye diseases, as well as damage to various organs such as nerve and kidney damage, leading to disabling and life-threatening conditions [1]. 

Worldwide, diabetes is a severe health problem. In 2019, 463 million people between the ages of 20 to 79 years were suffering from diabetes, and it is expected that this number will increase to 700 million in 2045. It is estimated that 4.2 million people died from diabetes in 2019. There is an increasing prevalence of diabetes by age and slightly less prevalence in women compared to men [2]. Whereas the raw prevalence of diabetes is the largest in the North American and Caribbean region (13.3%), the age-adjusted comparative prevalence of diabetes, which is more suited for comparing between regions and countries, is the highest in the Middle East and North Africa (12.8%) and the lowest in Africa (3.9%). The lower age-adjusted prevalence in Africa may be ascribed to a lower level of urbanization and to under-nutrition, resulting in lower levels of overweight and obesity. These numbers illustrate the enormous impact of diabetes on public health and the associated economic challenges. The total (worldwide) amount of health expenditure on diabetes was USD 760.3 billion in 2019, and the economic impact is expected to continue to grow [2]. Diabetes is no longer a problem for people in developed countries alone because diabetic cases in developing countries are also increasing. In 2019, in the African region, 19.4 million adults between 20 and 79 years were estimated to have diabetes (age-adjusted prevalence of 3.9%) and 45 million have developed Impaired Glucose Tolerance, which contains a high risk of developing type 2 diabetes (T2DM). About 366,000 deaths were attributed to diabetes; 3.1% of all deaths attributable to diabetes occurred in people under 60 years, which is the highest proportion in the world. There is an increased prevalence of diabetes in urban populations compared to rural populations. Specifically for Tanzania, one to two million of its fifty-six million citizens currently have diabetes, which corresponds to an age-adjusted prevalence of 5.7%. The number of diabetics in the African region is expected to increase by 143% by 2045 [2]. Undiagnosed diabetes is also the highest in the African region, which may be due to geographical constraints, limited resources, and the prioritization of other health issues. Mozambique has the greatest proportion of undiagnosed diabetes (86.7%), followed by Tanzania (79.8%) and Tunisia (75%). Actions focusing on the prevention of diabetes are becoming very urgent. Besides diabetes itself, diabetics often suffer from cardiovascular diseases (CVD) as well, resulting in higher mortality; more than 50% of people with diabetes die of CVD [3]. Therefore, it is clear that actions related to hygiene, food consumption, education, and health care are urgently required to prevent diabetes and associated diseases that subject a diabetic to high mortality risks. 

In Tanzania, about 60% to 70% of the urban population and up to 80% of the rural population use traditional medicines for their health care services because the majority cannot afford to purchase modern medicines and the national budgets for public health services are too limited to enable availability/accessibility of medicines to all [4]. These traditional medicines alone, and sometimes concomitantly with conventional medicines, are used in the treatment of diabetes [5]. African nature, and more precisely Tanzanian nature, is rich in different plant and insect species, and in Sub-Saharan Africa, the eating of insects is a common practice. Despite the fact that a good number of edible insects are known while some are also used traditionally for medicinal purposes, the research on their use for treating different diseases is scant. Exploiting various natural sources such as insects and plants by identifying and investigating how they can be used to manage and/or treat diabetes is essential to counter the increase in diabetic cases. Positive results would be of high value for the local people suffering from diabetes as they would benefit from locally available natural remedies, while developed countries might learn of new opportunities for treating or preventing diabetes. 

This was the outset of the ‘NAturals to prevent and/or cure DIAbetes (NADIA)’ project, a collaboration between the laboratories of the authors of this paper. The main goal of the NADIA project (VLIR-UOS; www.vliruos.be, accessed on 3 December 2021) is to develop an increase in competence and experience of academic staff in Tanzania to identify and investigate useful insects and plants to prevent and cure diabetes and to develop a long-term approach towards a better health situation (preventive and/or curative) for diabetic patients in Tanzania. In this review, the focus is on the potential use of insects in the battle against diabetes and associated complicating diseases.

## 2. Diabetes Mellitus

A distinction can be made between diabetes mellitus types 1 (T1DM) and 2 (T2DM). T1DM is an autoimmune disease; it occurs mainly in childhood and involves a complex interaction of genetic, immune, and environmental factors that destruct the function of the beta-cells. This form accounts for about 5–10% of people with diabetes [6]. T1DM is defined by the presence of autoimmune markers; however, there are some rare forms of T1DM that have no evidence of autoimmunity [7]. People suffering from T1DM can survive with daily insulin injections, blood glucose monitoring, physical activity, and a healthy diet.

T2DM is the most common type of diabetes and is linked to insulin resistance, i.e., the inability of the body’s cells to fully respond to insulin. T2DM is generally less severe than T1DM, occurs mainly in adulthood, and is usually caused by diet and lifestyle factors such as high carbohydrate/low fiber diets and a sedentary lifestyle. Genetic predisposition is also a contributing factor. [1] One-third to one-half of people with T2DM in the population may be undiagnosed, which can result in complications such as retinopathy that may fail to heal when diagnosis is finally made. T2DM can be managed by promoting a lifestyle that comprises a healthy diet, physical exercise, a healthy body weight, non-smoking, and, if necessary, medicines [2].

## 3. Diet and Diabetes

The etiology of T2DM is complex and involves interplays between several factors such as age, ethnicity, genetics, diet, physical activity, smoking, and awareness. Some of these factors can be influenced and reversed. Diet, in particular, is regarded as a main cause of T2DM among rich people who have consumed oil, flour, and sugar in large amounts since early in history. This is supported by the fact that, during the First and Second World Wars, mortality due to T2DM decreased in countries wherein famine existed but not in countries in which there was no shortage of food [3]. Poor diet, such as high carbohydrate/low fiber diets and sugar-rich beverages, combined with a sedentary lifestyle, causes obesity, which is associated with an increased incidence of diabetes. Studies show increased risks for developing diabetes in people with a BMI of ≥25 [8]. Due to low levels of physical activity, less glucose in the blood is taken up by the body to be utilized for energy production [1]. Fortunately, there is opportunity to reduce the incidence of T2DM by changing the diet and lifestyle of people with evidence of ‘prediabetes’ [8]. Prediabetes is characterized by evidence of an elevated blood sugar level that is not high enough to be considered T2DM. It is thus a condition between normal glucose regulation and T2DM, but people with prediabetes have an increased risk of developing T2DM and CVD [2].

Large scale clinical trials have shown that modest changes in diet and physical activity can reduce the incidence of T2DM by more than 50% for people with impaired glucose regulation [9]. In the Diabetes Prevention Program, subjects with impaired glucose tolerance were divided in an intensively guided lifestyle intervention group, a medication intervention group (with lifestyle recommendations), and a placebo group (with lifestyle recommendations). The largest reduction in risk for developing diabetes was observed in the intensive lifestyle intervention group [10].

Weight loss and physical activity are the main factors that drive the prevention of diabetes. However, the intervention programs that were used in the clinical studies were personalized to match the unique situation and cultural aspects of the participants [8]. Translation of the extensive interventions used in clinical trials to pragmatic but effective approaches in real life situations is challenging [11] and will require partnerships between health care systems that can adequately assess diabetes risk and community agencies that have the necessary resources to organize long-term lifestyle interventions [12].

As diabetes is associated with additional health problems such as CVD, interventions in diet can have a profound effect on these other conditions as well. Many studies have focused on the effects of diet on diabetes prevention and for ameliorating those suffering from T2DM and associated health problems. The data from meta-analyses suggest that nutrition therapy is powerful in the primary prevention of T2DM and associated risks [13], and several diets are acceptable to manage diabetes [14]. Classical nutritional approaches such as the low-fat diet (to prevent CVD) and the low-protein diet (to prevent diabetic kidney disease) have been questioned based on evidence, but low-carbohydrate diets or Mediterranean diets are advised [14]. The quality of dietary fats and carbohydrates is more important than their quantity in the diet. Diets that are rich in wholegrains, fruits, vegetables, legumes, and nuts; moderate alcohol consumption; and diets lower in refined grains, red or processed meats and sugar-sweetened beverages were shown to reduce the risk of diabetes and to improve glycemic control and blood lipids in diabetics [15].

Nutritional strategies are also important in managing patients with T2DM. In addition, research has been carried out on using diet supplements for glycemic control and reduction in risk for diabetes-related complications [16]. This includes the evaluation of plant extracts [13].

## 4. Traditional Medicine and Diabetes

Traditional medicine (also named indigenous or folk medicine) is defined as the sum of the knowledge, skills, and practices based on the theories, beliefs, and experiences indigenous to different cultures, whether explicable or not, that are used in the maintenance of health as well as in the prevention, diagnosis, improvement, or treatment of physical and mental illness [17]. In developing countries, a large proportion of the rural population still relies on traditional medicine for primary care. About 95% of the total components of traditional medicine are medicinal plants. Drugs currently used for the management of T2DM show several limitations, including adverse effects and lack of responsiveness, and medicinal plants may provide a source for the discovery of new drugs. Compared with modern medicine, herbal medicine can be accessed more easily by local people [4]. Research on botanicals (e.g., Cinnamomum species, *Xylopia aethiopica*, *Psidium guajava*) has shown their beneficial effects on T2DM and its complications [18,19].

In Tanzania, about 60% of the urban population and up to 80% of the rural population use traditional medicines for their health care services. Most of the traditional medicines are botanical products, and there are about 75,000 traditional medicine practitioners [20]. While traditional healers cannot always diagnose diabetes as a disease, some of the healers are well-informed of plants that are useful in alleviating the symptoms of diabetes. Some of the plants used are reported to have hypoglycemic and hypocholesterolemic activity and may thus prove to be useful in the management of T2DM [21].

## 5. Insects and Traditional Medicine

Although botanicals are mainly considered for use in traditional medicine in Tanzania, other sources such as insects and invertebrates may also be relevant. Here, we present an overview of the literature on the use of insects in entomophagy and entomotherapy.

### 5.1. Entomophagy

In Western society, eating insects is not compatible with current habits, and insects are far from accepted as a substitute for meat [22]. In many parts of the world, however, entomophagy is practiced and over 1900 species are known to be part of human diets [23]. The average energy content of insects is comparable to meat. Rumpold and Schlüter (2013) [24] published the nutritional values of 236 edible insects. From their work, the average nutritional composition of reported edible insects at the order level is presented in Table 1. The composition of edible insects is subject to large variation within and among species (and within and among orders) and is dependent on development stages, different nutritional intake, different origins, and, possibly, the different analytical methods used. Among insect orders, the average protein to dry weight content ranges roughly from 40% to 60%, which is higher than plant protein sources [25]. Average fat contents range from 12% to 35%; average fibers from 5% to 15%; average Nitrogen-Free Extract (NFE) from 5% to 20%; and average ash from 3% to 10%. The main fractions are the proteins followed by the fat fraction and the mean energy content ranging from 409 to 509 kcal/100 g, which is substantial [24]. Given the high protein content, several insect species potentially present an excellent alternative protein source, taking into account that protein quality and digestibility and amino acid profile are important factors. In general, all edible insect orders have amino acid levels (including essential amino acids) that meet WHO requirements [24]. Fat is the second largest fraction and its content is highly variable between insect orders and species; larvae and pupae have more fat than adult insects [25]. The fraction of saturated fatty acids (SFA) ranges on average from roughly 30% to 42% and mainly comprises palmitic acid and stearic acid. Noteworthily, the black soldier fly, an insect commonly reared but not considered for food applications, is very rich in lauric acid (~50%) [26]. The mean fraction of monounsaturated fatty acids (MUFA, including oleic acid and palmitoleic acid) ranges from 22% to 48% and the average amount of polyunsaturated fatty acids (PUFA) varies from 16% to 40%. The main PUFA comprise linoleic acid, α-linolenic acid, and γ-linolenic acid. Arachidonic acid and eicosapentaenoic acid are reported in some species as well [24]. The fatty acid profile can vary with the diet of insects and can thus be tailored to some extent [27]. Cholesterol content can be influenced by the insect diet [28] and can range from 7 mg/100 g dry sample to up to 100 mg/100 g dry sample [24]. 

Mineral and vitamin composition are also important for the nutritional value of edible insects; however, large variations (among and within species and orders) are observed in published numbers [24]. In general, insects do not seem to fulfil daily requirements of calcium and potassium, and they are also low in sodium. Availability of other minerals depends on the species. Edible insects may have the potential to provide micronutrients such as copper, iron, magnesium, manganese, phosphorus, selenium, and zinc, but little is currently known of their bioavailability [29]. With the exception of some more frequently eaten insects, less information is available on the vitamin content of edible insects and this can be highly dependent on the species [24]. There are data published that indicate that carotene and vitamins B1, B2, B6, C, D, E, and K occur in edible insects [25].

More than 2 billion people worldwide, mostly in Africa, Asia, Latin America, and Oceania, include various species of insects in their diet [25,30]. In developing countries, entomophagy is encouraged because animal proteins are expensive for the underprivileged populations and an increase in entomophagy could relieve malnutrition and decrease the pressure exerted on other protein sources. The expected increase in the human population in the near future, the effects of global warming on the reduction in areas used for food production, and the environmental destruction due to industrial development all indicate a resource shortage, and alternative food sources are needed [25]. Moreover, in developed countries, the potential of insects as a sustainable, nutritious, alternative source of protein is increasingly recognized, shown by an increase in economic activities on insect research, rearing, and implementation in food and feed [31]. 

Africa is a very important source of edible insect biodiversity in the world [32]. More than 500 insect species are used as food, and insects are important for tradition, medicine, and diet. Insects are eaten as a daily supplement, an occasional dish, or a substitute product during food shortages [33,34]. The most consumed insects in Africa are caterpillars and termites, but other species (e.g., locusts) can be locally important for economic, ecological, or nutritional reasons [33,35]. In many instances, the insects are gathered from bushes and farmland, processed, and eaten or sold on local and urban markets or exported to cities [23].

Whereas many studies suggest that the consumption of edible insects promotes desirable health outcomes, caution is also warranted as sensitive people may have allergic reactions in response to eating insects [30].

### 5.2. Entomotherapy

Throughout history, insects and other arthropods and products thereof have been used worldwide for medical application [36]. Whereas in Western society and more economically robust countries conventional medical treatments are preferred, the use of traditional medicine is still practiced in many parts of the world, including India, Korea, China, South America, and Africa [37,38]. While traditional medicine practices are more integrated and documented in Chinese and Korean culture, they are less well-documented for African regions [39]. However, in African regions where access to modern medicine is limited, traditional medicine, including the use of insects, is an important practice. Since the rise of modern medicine and due to the often strange instructions on how to perform procedures, traditional medicine has often been dismissed as superstition or nonsense [40]. Many of the traditional medicinal uses of insects are dismissed as being superstitious. In different cases, this is correctly so; nevertheless, some of even the most incredulous practices could possess merit [36]. Whereas in Western society the use of plants for medicinal applications seems to be more acceptable, records exist dating to ancient times that contain information on the medicinal use of insects and, in Europe, traditional healers have used insects medicinally [36].

The earliest records date back to the Egyptian Ebers papyrus of the 16th century BC that contains several accounts of medicines derived from insects and spiders. Extracts of silkworms have been used in Chinese traditional medicines for over three thousand years and living larvae of certain flies have been used worldwide for centuries to treat infected wounds [40]. Furthermore, in Europe, it was believed that insects had healing powers. Oil extracted from May beetle larvae was believed to cure rheumatism and pulverized cockroaches were used as treatment for epilepsy. The literature indicates that the number of arthropod species and other invertebrates used medicinally outnumbers the species used as food. However, in contrast, searches also indicate that the vast amount of contemporary literature involves the use of insects for food and not on their therapeutic implementation [36]. Thus, although entomotherapy is an ancient practice making use of numerous arthropod and invertebrate species, its use has been strongly reduced due to the advance of medical science [40].

Aside from being a good source of protein and thus an important component of a healthy diet, the use of insects in diets has been implemented for specific health effects, including diabetes and associated health complications such as CVD. Cultures that consume insects also associate them with several health benefits beyond nutrition [25], such as the Chinese caterpillar fungus that is believed to have immunostimulatory and anticancer properties [41], or evidence that suggests that termites have immunostimulatory effects [41]. The exoskeleton of insects mainly consists of chitin, a macromolecular compound that is part of the carbohydrate fraction. In some studies, chitin has been shown to reduce serum cholesterol [42], a finding not always replicated in studies [43]. It has been shown in the literature that the consumption of PUFA has several health benefits, such as improved cognitive development and the reduction in glucose tolerance, thereby reducing the risk of diabetes, lowering blood pressure, and preventing insulin resistance [23,30]. Insect fats have been shown to be rich in polyunsaturated fatty acids such as linoleic and α-linolenic acids [23,33], which may beneficially influence these conditions. Diets with a high amount of unsaturated fatty acids may be used to prevent cardiovascular diseases that are associated with diabetes, and this suggests that the use of several insect species in diets may have potential for the management of certain coronary heart diseases [33]. Another insect that is historically believed to have beneficial effects on ameliorating T2DM is the bug *Thasus gigas*, which is commonly eaten in Mexico. However, its exact impact on diabetes has not yet been investigated in a properly designed scientific study [44].

Due to sheer numbers, it is impossible to describe in detail the different insect species and the ways in which insects are used in traditional medicine worldwide. However, the reviews of Costa-Neto (2005) [40] and Meyer-Rochow (2017) [36] shed light on this domain. As described in [40], of the 411 medicinal insect species recorded worldwide, 92.6% are used to treat 353 different disorders, including skin, digestive, respiratory, kidney, reproductive, circulatory, nervous, eye, neuromuscular, bone, immunological, hearing, and endocrinological diseases.

About 29 insect orders exist, but 80% of all insects belong to one of the following orders: Coleoptera, Diptera, Hymenoptera, and Lepidoptera [38]. In Table 2, a general overview is shown of the main insect orders used in traditional medicine. The examples (not exhaustive) of the diseases treated are taken from [36,38,40] 

One of the most well-known and well-studied medical applications is the use of maggots for wound healing. Living fly larvae are put on a wound and aid in the healing process by promoting fibroblast aggregation and tissue repair, eating necrotic tissue that otherwise could promote infection, releasing antibacterial substances, and destroying ingested bacteria. Larval therapy has been implemented since the Middle Ages and has been practiced by traditional healers in Asia, South America, Europe, and Australia [37]. In a trial involving diabetic leg ulcers, wounds treated with the larvae had significantly less necrotic tissue after two weeks compared to conventional treatment, indicating that larval treatment can be safe and effective [45]. A review of the literature indicates that the use of maggots in wound healing may be appropriate in parts of the world where conventional therapies are not possible [37]. This maggot treatment is fairly well-known to the layman in Western society, however, a large number of other examples on medicinal implementation of insects can be found in the literature (Table 2). The venom from bees, wasps, and ants has been used in traditional medicine to treat arthritis, rheumatism, skin diseases, multiple sclerosis, cancer, infections, and pain [37]. Some wasp venoms have been shown to contain more than 75 different components [46]. One of the molecules on which research focuses is melittin, a peptide from honey bee venom that shows potential as a therapeutic medicine for treating different types of cancer. Another insect-derived molecule that is being explored for the treatment of cancer is cantharidin, a defense toxin of so-called blister beetles that causes skin blistering upon contact. The powder of dried beetles is also known as ‘Spanish Fly’ as it is supposed to have aphrodisiac properties as well [47]. Based on this molecule, norcantharins have been produced that still have anticancer properties but are less toxic [48]. Dried bodies and secretions of insects and extracts thereof have been widely used in traditional medicine for treating infections. Insects have developed robust immune defenses under the form of antimicrobial peptides (AMPs), which have great potential for combatting the antibiotic-resistant pathogens. Despite the research already performed, limited clinical applications have yet been developed for use as antimicrobial products, possibly due to limited interest from large pharmaceutical companies in antimicrobial products in the past, the high production costs of AMPs, concerns about the stability and toxicity of AMPs to mammalian cells, and the development of bacterial resistance to the AMPs [47].

Insects are specialized in the synthesis of chemical compounds that function as pheromones, defensive sprays, venoms, and toxins [49]. These chemicals include compounds that are emetic, vesicant, irritating, cardioactive, or neurotoxic. As indicated in Table 2, many insects are used in the treatment of a diverse spectrum of diseases. Insects are used in dried pulverized form, or extracts from insects are used in the treatment regime, and they have been reported to ameliorate disease phenotypes. Considerable progress has been made in isolating the active chemicals from these insect extracts and evaluating them pharmacologically. The presence of proteins, terpenoids, sugars, polyols and mucilages, saponins, polyphenolic glycosides, quinones, anthraquinone glycosides, cyanogenic glycosides, and alkaloids has been confirmed in several extracts from insects [38]. In Table 3, a selection of small chemicals that have been isolated from insects and which are investigated for potential therapeutic uses are presented.

All examples derive from [38], which can be consulted for specific chemical structures.

### 5.3. Potential of Entomotherapy in the Combat against T2DM and Related Complicated Disorders

Regarding their potential to combat T2DM and related complicated disorders, some insects and insect-derived products have been described that may also have medicinal applications. Examples are shown in Table 4 and are explained in more detail in the text.

Diagnosis of diabetes in some tribes in Africa is made when ants are seen to feed on a person’s urine [50]. As already mentioned, Nallely et al. (2014) [44] explored the effect of ingestion of xamues (*Thasus gigas*) as an alternative treatment for T2DM in Mexico. These insects are consumed and are believed to ameliorate diabetes, but there are no objective data available that indicate this effect. The study of Nallely et al. indicates that some people actually consume the insect as a treatment for diabetes, and 7% of people with T2DM have discontinued their allopathic treatment [44]. It is thus important to conduct further research on the relationship (or lack of) between consumption of xamues and glucose level in blood. Similarly, Argentinian people consume beetles (*Ulomoides dermestoides*) as an alternative medicine to treat illnesses, including diabetes [51].

A molecule that may be useful in combating CVD complications is chitin, an aminopolysaccharide that makes up the exoskeleton of insects. Chitosan, a deacetylated form of chitin, has been shown to have a total and an LDL cholesterol lowering effect in animals and humans. The mechanism for this effect is not yet known, but as little as 1.2 g per day may provide significant reductions in serum cholesterol. Studies suggest that chitosan could accelerate human weight loss if used in combination with a weight reduction diet [52]. Similarly, chito-oligosaccharide, derived from enzymatic or chemical hydrolysis of chitin, has also been shown to have suppressive effects on hyperlipidemia and hypercholesteremia [63]. Chitin could thus play a role in the prevention of atherosclerotic diseases such as coronary heart disease and stroke, which are prevalent complications for patients suffering from T2DM. Therefore, chitin is an example of a product derived from insects that may have some bioactivity against a T2DM complication.

Anticoagulants from leeches and ticks have been investigated for potential use as an anticlotting agent, but insects also contain interesting substances, an example being anopheline, found in the salivary glands of Anopheles mosquitoes, which has an unique thrombin inhibition mechanism that has potential for the design of novel antithrombotics [53].

Recently, the anti-diabetic and anti-obesity effects of functional, active proteins obtained from edible insects were investigated [54,55]. Several bioactive peptides (BAPs) were identified in protein hydrolysates from insects [64]. Dipeptidyl peptidase-IV (DPP-IV) is an enzyme that is involved in the inactivation of incretins, which are gut-derived hormones that play a role in glycemic regulation. Inhibition of the DPP-IV enzyme by gliptins is a strategy that is used in the treatment of T2DM [65]. Protein hydrolysates from edible insects have indicated the presence of BAPs with in vitro DPP-IV inhibiting capacity, thus showing the potential of insect protein hydrolysates as a functional food ingredient to improve glycemic regulation [54,55]. Similarly, BAPs were identified in insect protein hydrolysates that inhibit α-glucosidase and α-lipase. α-Glucosidase is a metabolic key enzyme in the gut during the post-prandial phase that cleaves oligosaccharides into glucose. Inhibition of this enzyme can thus reduce post-prandial hyperglycemia in T2DM. α-Lipase is involved in the breakdown of dietary fats into glycerol and free fatty acids in the digestive tract; inhibiting this lipase by BAPs can thus reduce lipid absorption and can be used to treat obesity [54].

Bee venom has been reported to have good anti-diabetic effects by increasing the insulin level in mouse models [56]. In a small study, the effect of bee stings on lowering the blood glucose level in humans was demonstrated [38,57]. In addition to glucose, triglycerides and cholesterol were also reduced. Melittin, which is a major component of bee venom, has been shown in the past to stimulate the pancreatic beta-cells to increase insulin secretion [58]. Additional possible mechanisms for modulating diabetes may involve the stimulation of phospholipase A, an increase in glucose uptake, improving lipid profile, and a reduction in inflammation [59].

Diabetic nephropathy is a major complication of diabetes. Sustained hyperglycemia can increase the production of free radicals, which can result in oxidative stress, inflammation, and fibrosis in the kidneys [66]. Wang et al. [60] made an ethanolic extract rich in flavonoids and flavonoid glycosides from the silkworm green cocoon shell and evaluated its effect on T2DM mice. The ethanol extract administration resulted in lower blood glucose levels and in reduced body weight in diabetic mice compared to the negative control mice. The results indicated that ethanolic extracts may reduce oxidative stress and deactivate phosphorylation of p38 MAPK in T2DM mice, which may decrease the secretion of inflammatory cytokines and reduce the occurrence of fibrosis in diabetic nephropathy. The ethanolic extract may thus be useful as a natural medicine for diabetic nephropathy [60]. In rabbits, a similar extract was found to have significant effects on hypercholesterolemia and atherosclerosis through a lipid-lowering capability and by decreasing the extent of atherosclerotic lesions [61].

The biologically active products are not always synthesized by insects, but instead can sometimes be traced back to associated microorganisms or to plants and even animals [62]. This is illustrated by a cholesteryl ester transfer protein inhibitor (CETPI), which is derived from a Cytospora fungus that is associated with *Pogonomyrmex badius* ants and which may reduce the risk of coronary heart disease by raising HDL and lowering LDL levels at the same time [62].

## 6. Perspectives on the Use of Insects to Prevent and/or Cure Diabetes in Tanzania

In Tanzania, and more generally in sub-Saharan Africa, the prevalence of diabetes is rising, and about 60% of adults aged 20–79 years with diabetes are undiagnosed. As urbanization increases and populations age, T2DM will pose an increasing problem [2]. As expensive therapies and access to medication are more limited, use of traditional medicines and prevention through healthy diets may provide a strategy to limit the numbers of T2DM in the future. As described in this overview paper, insects may play a role in this context via entomophagy and entomotherapy approaches.

Given that T2DM is associated with obesity and an unhealthy diet, one approach to battle T2DM and associated disorders such as cardiovascular diseases (CVD) may involve a prevention campaign that implements insects in healthy diets. As shown earlier, insects contain a large proportion of proteins with essential amino acids and the insect fats contain a large fraction of PUFA and fibers. By integrating selected nutritious insects into balanced diets, healthy food may be provided to battle obesity, CVD, and the potential development of T2DM.

Between 1961 and 2010, 939 nature-derived approved drugs were developed; none of these were from insects, but rather from plants, marine organisms, microbes, and invertebrates such as leeches [47]. However, due to their enormous diversity, insect species (>1 million species) and other arthropods are a large source base for the discovery of natural products [62]. Insects are specialized in the synthesis of chemical compounds that function as pheromones, defensive sprays, venoms, and toxins [49]. Insects thus present a potential source for the discovery of novel molecules that may be used as a starting point for the development of therapeutic molecules that can be used to treat T2DM and related disorders, and several examples have been presented in this paper.

To conclude, the geographical location and climate of Africa favors the rich flora and fauna that includes large quantities of insects. In some African countries, insects are eaten, but research on their use for treating different diseases is rare. Only a few studies on insects have been carried out regarding diabetes, and some insect products exhibit antidiabetic activity as well as having bioactivities relevant to the control of risk factors/disorders of diabetes. Future focused and detailed studies of both insects and plants could provide safe standardized natural remedies for diabetes that would be of high value for the local people as the majority are not able to afford modern antidiabetic medicines. In addition, developed countries might learn of new opportunities for treating or preventing diabetes and collaborative scientific studies could lead to the isolation and identification of bioactives, which may lead to more effective medicines with less/few side effects compared to the current antidiabetic medicines.

In this review, we attempted to present the perspectives on the implementation of insects as a nutritional preventive and/or as a source for curative approaches in the battle against diabetes and associated complex disorders. 

## Figures and Tables

**Table 1:** Nutritional composition (%) and energy content (Kcal/100 g) of different orders of edible insects (dry weight).

Edible Insect	Protein (%)	Fat (%)	Fiber (%)	NFE (%)	Ash (%)	Energy Content (Kcal/100 g)
*Blattodea* (cockroaches)	57.30	29.90	5.31	4.53	2.94	/
*Coleoptera* (beetles, grubs)	40.69	33.40	10.74	13.20	5.07	490.30
*Diptera* (flies)	49.48	22.75	13.56	6.01	10.31	409.78
*Hemiptera* (true bugs)	48.33	30.26	12.40	6.08	5.03	478.99
*Hymenoptera* (ants, bees)	46.47	25.09	5.71	20.25	3.51	484.45
*Isoptera* (termites)	35.34	32.74	5.06	22.84	5.88	/
*Lepidoptera* (butterflies, moths)	45.38	27.66	6.60	18.76	4.51	508.89
*Odonata* (dragonflies, damselflies)	55.23	19.83	11.79	4.63	8.53	431.33
*Orthoptera* (crickets, grasshoppers, locusts)	61.32	13.41	9.55	12.98	3.85	426.25

NFE = nitrogen-free extract, i.e., carbohydrates (NFE = 100% − (protein + crude fat + ash + crude fiber + moisture). Numbers are derived from [24].

**Table 2:** General overview of insect orders and their use in traditional medicine (Adapted from [36,38,40].

Insect Order	General Examples	Selection of Diseases Treated
*Hymenoptera*	Bees, wasps, ants	Rheumatic pain, arthritis, headache, haemorrhoids, mumps, asthma, dizziness, colds, paralysis, ulcers, acne
*Coleoptera*	Beetles	Kidney pain, rheumatism, ear and tooth aches, wound healing, hair growth
*Blattodea*	Cockroaches	Regulate menstruation, control urination, renal colics, asthma, stop bleeding, heal bone fractures, remove swellings
*Phasmida*	Walking stick	Asthma, upset stomach, muscle pain
*Diptera*	Flies, mosquitoes	Eye cysts, baldness, wound healing, osteomyelitis
*Hemiptera*	True bugs	Goiter, tuberculosis, cough, skin disease, liver, stomach, and kidney diseases
*Lepidoptera*	Butterflies, moths	Asthma, earache, hemorrhage after delivery, shortness of breath, weak kidneys, impotence
*Orthoptera*	Grasshoppers, crickets, katydids	Kidney, ulcerating, and fever complaints, venereal diseases, urinary problems, mental issues, wound healing, liver disorders, anaemia, dental caries
*Isoptera*	Termites	Asthma, hoarseness and sinusitis, wounds, malnutrition, heart conditions, anaemia, anti-diarrhoea, tuberculosis, prevent miscarriages

**Table 3:** Insect-derived small molecules and their potential therapeutic use.

Species	Molecule	Examples	Use
*Hymenoptera*: sawfly	Phloroglucinol derivatives	Macrocarpal, grandinol	Antimicrobial action
*Hymenoptera*: iron ants	Tetraponerines	Several structures identified	Cytotoxicity against tumor cell lines
*Hymenoptera*: fire ants	Alkaloids	Solenopsin A	Antiangiogenic activity by selective inhibition of protein kinase B (AKT) cancer treatment
*Hymenoptera*: red ants	Alkaloids	N-(2-hydroxyethyl)-benzamide	Antibacterial action
*Hymenoptera*: Chinese black ants	Dopamine derivatives	Polyrhadopamines, trolline, etc.	Treatment for cardiovascular, neurological, oncological, and renal diseases; rheumatoid arthritis
*Hymenoptera*: Chinese black ants	N-containing molecules	5-(3-indolylmethyl)-nicotinamide, β-carboline-3-carboxamide, 3-hydroxypyridine, etc.	Rheumatoid arthritis therapy, kidney problems, anti-inflammatory action
*Hymenoptera*: wasp	Polybiosides	Polybiosides α and β	Neuroactive effect/stimulation of neurons
*Catharsius* molossus	N-acetyldopamine dimers	Molossusamides A, B, C	Anti-inflammation
*Coleoptera*: tenebrionidae	Phenolics	Blapsols A-D; dopamine dimers	Treatment of pathogenic inflammation by inhibiting COX enzymes
*Coleoptera*: bruchidae larvae	Lipids	Dorsamin-A763, A737, A765, A739, A767	Antioxidant effects
*Coleoptera*: Meloid beetles	Terpene-related compounds	Cantharidin, norcantharidin, 5,6-dehydronorcantharidin, hydroxycantharidinimide	Derivatives with less toxicity are used in tumor therapy; potential use as vasoconstrictor and positive inotrope for cardiac failure; treatment of warts and molluscum
*Blattodea*: *P. americana*	Isocoumarins	Periplatins A-D	Cytotoxic to cancer cells; improvement in patients with sepsis
*Blattodea*: *Polyphaga plancyi*	Phenolics/N-containing compounds	Plancyols A and B, plancypyrazine A, plancyamide B	Treatment of cancer
*Hemiptera*: Chinese stinkbug	N-acetyl-dopamine derivatives	Aspongamide A, aspongopusamides A-D	Chronic kidney disease; COX2 inhibition
*Hemiptera*: Chinese stinkbug	Sesquiterpenoids and other small molecules	Aspongnoids A-D, asponguanines A-D, aspongadenines A-B, aspongpyrazines A-B, aspongester A	Promotion of proliferation of neural stem cells
*Orthoptera*: Texas grasshopper	Alkaloids	Pancratistatin, narciclasine, ungeremine	Anticancer agents
*Lepidoptera*: Taiwan butterfly	Phenanthrene dicarboxylic acid	Papilistatin	Cytotoxicity against pancreatic cancer cells

**Table 4:** Overview on the medicinal use of insects and derived products for diabetes or associated complicating disorders.

Insect	Substance	Disorder	Mechanism	Reference
Ants	/	Diabetes	Diagnostic value if ants feed on a person’s urine	van Huis, 2002 [50]
Xamues (*Thasus gigas*)	Whole insect	Diabetes	/	Nallely et al., 2014 [44]
Beetles (*Ulomoides dermestoides*)	Whole insect	Diabetes	/	Crespo et al., 2011 [51]
All insects	Chitin/chitosan	CVD	Total and LDL cholesterol lowering effect	Gallagher, 2003 [52]
Anopheles mosquitoes	Anopheline	Cardiac disease	Antithrombotics	Figueiredo et al., 2012 [53]
Lesser mealworm (*A. diaperinus*)	Bioactive peptides	Diabetes, obesitas	Inhibition of DPP-IV	Lacroix et al., 2019 [54]
Cricket, locust, silkworm, bamboo worm, house fly, mealworm, weaver ant	Bioactive peptides	Diabetes, obesitas	Inhibition of α-glucosidase and α-lipase	Matheswaran et al., 2020 [55]
Bees (*Apis mellifera*)	Venom	Diabetes	Increase in insulin level	Mousavi et al., 2012 [56]
Bees (*Apis cerana*)	Bee stings	Diabetes	Lowering blood glucose, triglyceride, and cholesterol levels	Prakash and Bhargava, 2014 [57]; Seabrooks and Hu, 2017 [38]
Bees	Mellitin	Diabetes	Stimulation of pancreatic beta cells to increase insulin secretion	Morgan et al., 1984 [58]; Hossen et al., 2017 [59]
Silkworm cocoon	Ethanol extract	Diabetic nephropathy	Reduction in oxidative stress and fibrosis	Wang et al., 2019 [60]
Silkworm cocoon	Ethanol extract	Hypercholesterolemia and atherosclerosis	Lipid lowering capability and lowering extent of atherosclerotic lesions	Ali and Arumugam, 2011 [61]
Ants (*Pogonomyrmex badius*)	Cholesteryl ester transfer protein inhibitor	CHD	Raises HDL and lowers LDL	Dettner, 2011 [62]

## Data Availability

Data sharing not applicable.
